# Aortitis requiring aortic repair associated with glaucoma, thyroiditis, glaucoma, and neuropathy: case report

**DOI:** 10.1186/1749-8090-6-74

**Published:** 2011-05-16

**Authors:** Claudia Stöllberger, Marion Avanzini, Aileen Hanafin, Ramona Sanani, Georg Wieselthaler, Nikolaus Wick, Günther Bayer, Günther Mölzer, Josef Finsterer

**Affiliations:** 1 2nd Medical department, Krankenanstalt Rudolfstiftung, Juchgasse 25, Wien, A-1030, Österreich, Austria; 2 Department of Gynecology and Obstetrics, Krankenanstalt Rudolfstiftung, Juchgasse 25, Wien, A-1030, Österreich, Austria; 3 Department of Cardiac Surgery, Allgemeines Krankenhaus, Währingerstraße 18-20, 1090 Wien, Österreich, Austria; 4 Institute of Pathology, Allgemeines Krankenhaus, Währingerstraße 18-20, Wien, A-1030, Österreich, Austria; 5 Department of Radiology, Krankenanstalt Rudolfstiftung, Juchgasse 25, Wien, A-1030, Österreich, Austria; 6 Department of Neurology, Krankenanstalt Rudolfstiftung, Juchgasse 25, Wien, A-1030, Österreich, Austria

## Abstract

Aortitis may be due to infectious and non-infectious causes. We observed aortitis, associated with glaucoma, thyroiditis, pericarditis, pleural effusion and neuropathy in a 63-years old woman. Despite antibiotic therapy, inflammatory signs persisted and resolved only after initiation of glucocorticoid therapy. Increasing aortic ectasia necessitated resection of the ascending aorta and implantation of a Vascutek 30 mm prosthesis. Histologically a granulomatous aortitis was diagnosed. Since all other possible causes were excluded, an immunological mechanism of the aortitis is suspected and possible triggering factors are discussed.

## Background

Aortitis of the ascending aorta may be due to infectious and non-infectious causes, comprising several systemic disorders [1,2]. Aortitis, associated with glaucoma, thyroiditis, pericarditis, pleural effusion and neuropathy with prompt clinical response to glucocorticoids has not been described so far.

## Case presentation

A 63-year old HIV-negative Iranian female was hospitalized in May 2004 because of fatigue, undulating fever, night sweats, and weight loss starting 8 weeks before. In 1991 and 1992 she had undergone bilateral iridectomy because of glaucoma. She has had 2 uneventful pregnancies and wan through menopause at the age of 52. Twenty-seven years ago, an intrauterine device (IUD) had been implanted but never been removed. She smoked 20 cigarettes/day.

**Figure 1:**
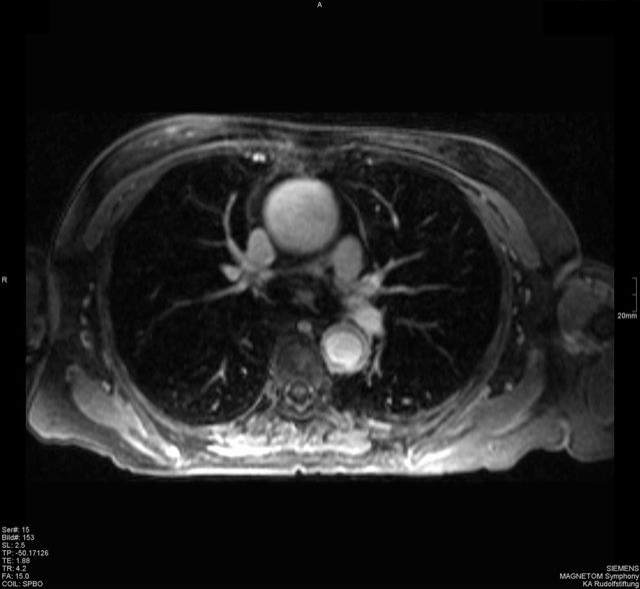
**Thoracic CT from May 2004 - Thoracic CT from May 2004 showing the ectatic ascending aorta with a diffuse wall thickening**.

Physical examination was normal, the blood-pressure was 130/75 mmHg bilaterally. Blood tests showed normochromic anaemia (haemoglobin 10.1 g/dl, normal >12.0), elevated CRP levels (12.3 mg/dl, normal <0.6) and an increased erytrocyte sedimentation rate (110 mm, normal <20). Blood cultures remained negative. Stool culture did not grow any pathogens. Antinuclear antibodies, their subsets and antineutrophilic cytoplasmic antibodies were negative. Antibodies against smooth muscle cells were repeatedly positive. Serologic tests for *Treponema pallidum*, *Brucella abortus*, *Brucella melitensis*, *Francisella tularensis, Yersinia enterocolitica, Yersinia pseudotuberculosis, Bartonella henselae, Borrelia burgdorferi, Coxiella burnetti, Ehrlichia, Leptospira, Mycoplasma pneumoniae *and *Ureaplasma urealyticum *were negative. Agglutination reaction with *Salmonella-H *unspecified was 1: 320 and with *Salmonella-HD *1: 1280. *Chlamydia SP*-IgA and IgG Antibodies were repeatedly positive. HLA-B27 testing was negative, while an HLA-DR4 15(2) allele was assessed. Computed-tomography (CT) and magnetic-resonance-imaging (MRI) of the chest showed an ectatic ascending aorta (50 mm) with diffusely thickened walls (Figure 1). She received an empirical antibiotic therapy with cephazolin and metronidazole for 9 days, and CRP decreased to 7.9 mg/dl. Hashimoto's thyroiditis was diagnosed and 100 μg/d levothyroxine was started.

**Figure 2:**
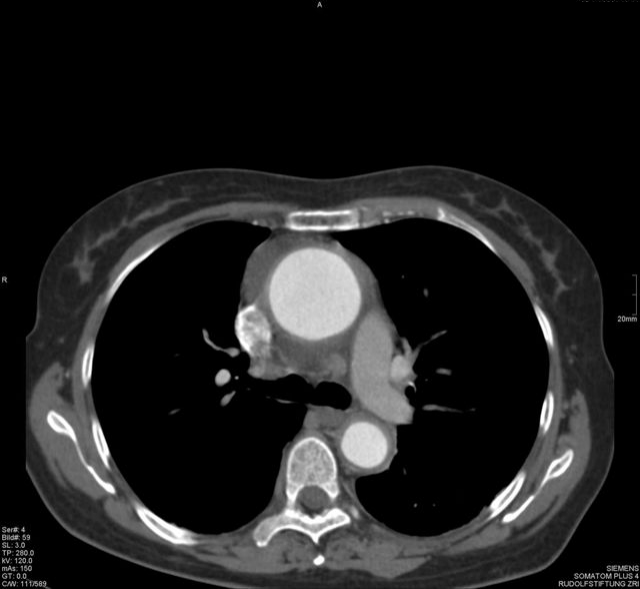
**Thoracic CT from August 2004 - Thoracic CT from August 2004 showing an increase in the thickness of the aortic wall**.

Two months later, she was again hospitalized because of chest pain, fever and dyspnoea. Echocardiography and chest CT detected 3 mm pericardial effusion and an increase in the thickness of the aortic wall from 10 to 16 mm (Figure 2). Since there were no signs of aortic dissection or an intramural haematoma, aortitis was assumed. Arterial hypertension necessitated pharmacological therapy. Cephazolin, and later piperacillin-tazobactam were started, without any effect on the elevated CRP levels (20.5 mg/dl). Eventually, bilateral pleural effusions necessitated drainage. The pleural fluid showed a glucose content of 71 mg/dl (⊥ < 60), total protein of 4.0 g/dl (⊥ 0.3 - 4.1) and a LDH activity of 302 U/l (⊥ < 200), but no growth of bacteria or *Mycobacterium tuberculosis*. Cytological investigation of the pleural fluid revealed inflammatory cells comprising granulocytes, lymphocytes, nuclear fragmented macrophages and degenerated mesothelial cells with cytoplasmatic inclusions. A biopsy of the left temporal artery revealed no signs of inflammation. Clinical and electrophysiological neurological investigations showed sensorimotor polyneuropathy of both lower limbs and she also displayed symptoms of depression.

Antibiotic therapy was stopped after 4 weeks, and 62.5 mg oral prednisolone was initiated. The patient's condition improved within a few days. The red cell blood count returned to normal values without transfusion of packed blood cells, the CRP levels and erythrocyte sedimentation rate nearly normalized. She was discharged with 47.5 mg metoprolol, 20 mg lisinopril, 25 mg hydrochlorothiazide, 4 mg doxazosin, 100 μg levothyroxine, 100 mg acetylsalicylic acid, 10 mg citalopram, 30 mg mirtazapine, 40 mg pantoprazol, 3 mg bromazepam and 25 mg prednisolone.

**Figure 3:**
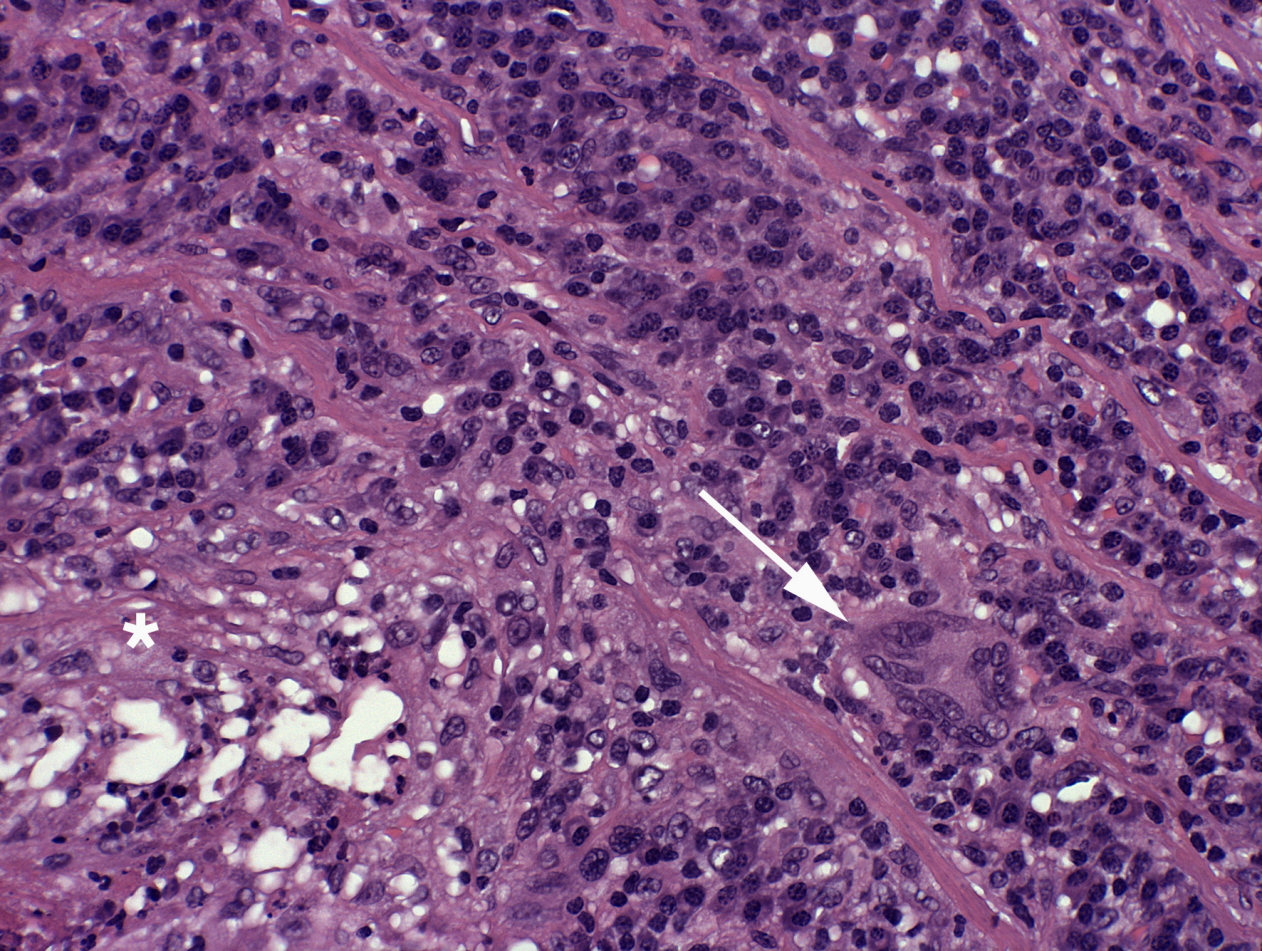
**Histologic picture of the resected aortic wall - After formalin-fixation and paraffin embedding a 2 μm transversal full section of the ectatic aortal segment was stained with H&E according to a standard protocol**. A dense and focally destructive inflammatory reaction that covered 75% of the wall thickness and predominated the media was observed. Specifically, focal necroses with neutrophilic granulocytes (asteriks, lower left) and bands of lymphocytic infiltrates with occasional multinuclear giant cells (arrow) could be identified. Magn.: 200 ×.

In November 2005 the diameter of the ascending aorta had increased to 55 mm, therefore the patient underwent resection of the aneurysm of the ascending aorta, implantation of a Vascutek 30 mm prosthesis and a thrombendarterectomy of the right subclavian artery. Histological examination of the resected aortic wall showed a granulomatous aortitis (Figure 3). Molecular genetic testing for *Mycobacterium tuberculosis *was negative. Prednisolone was stopped in January 2007. Since then, the patient is well, there are no clinical, biochemical, or haematological signs of inflammation, and she is currently managed with 100 mg metoprolol, 20 mg lisinopril and 30 mg mirtazapine. The IUD is still in place because it is impossible to remove it transvaginally.

## Discussion

Non-infectious aortitis is a rare cause of aortic aneurysm. In one major study, it was detected in 9% of 513 patients with surgically resected aortic aneurysms, and more frequently in females than in males [1]. Non-infectious aortitis may be due to Takayasu's disease, giant cell arteritis, spondylarthropathy, Behcet's syndrome, relapsing polychondritis, Cogan's syndrome, retroperitoneal fibrosis, ankylosing spondylitis, systemic lupus erythematodes, scleroderma, psoriasis, ulcerative colitis, Crohn's disease, radiation, Reiter's syndrome, or Wiskott-Aldrich syndrome [1,2]. In our case however, all the above mentioned diseases were excluded clinically or by laboratory testing.

Though the patient responded well steroid therapy, an infectious aetiology of the disease could not completely be excluded. Infectious aortitis results from septic embolization to the vasa vasorum, hematogenous seeding of an existing aneurysm, or extension from a contiguous site of infection, especially in patients with atherosclerosis [2]. Infectious aortitis may be due to *Streptococcus pneumoniae*, group A *streptococci, Haemophilus inflenzae, Staphylococcus aureus*, *Salmonella species*, *Pseudomonas*, *Treponema pallidum*, *Mycobacterium tuberculosis, Capnocytophaga canimorsus *or *Pasteurella multicida *[3-5]. However, the clinical presentation and laboratory findings did not indicate an infectious aetiology: Neither did she suffer from atherosclerosis nor was there any evidence of a contiguous site of infection.

It can be speculated that a potential site for a chronic infection or an immunologically mediated aortitis might be the IUD, which are known to increase the risk of pelvic inflammatory disease [6]. Most pelvic inflammatory diseases, specially *Chlamydia trachomatis *infections are asymptomatic [7]. The repeatedly positive serologic findings for *Chlamydia sp*. might indicate clinically silent pelvic inflammatory disease. Furthermore, IUDs are known to influence immune responses [8]. Antibodies against smooth-muscle cells, which were repeatedly positive in our patient, are considered diagnostic markers for autoimmune hepatitis. However, they are also a frequent finding in the sera of apparently healthy females [9]. It can be hypothesized, that the device within the uterus, an organ consisting of smooth muscle cells, might have stimulated antibody production. The benign clinical course during 5 years after aortic surgery, however, indicates that the pathogenic role of the IUD might not continue over a long time.

## Conclusions

This case shows that in aortitis, despite extensive investigations, occasionally no definite aetiology can be found. Even if regression of symptoms and inflammatory signs occurs after initiation of corticosteroid therapy, follow-up is warranted because progression of aortic ectasia could indicate a need for vascular surgery.

## Consent

"Written informed consent was obtained from the patient for publication of this case report and any accompanying images. A copy of the written consent is available for review by the Editor-in-Chief of this journal."

## List of abbreviations

CRP: C-reactive-protein; HIV: Human immunodeficiency virus; IgA: Immunoglobuline A; IgG: Immunoglobuline G; IUD: intrauterine Device; LDH: Lactate-dehydrogenase;

## Competing interests

The authors declare that they have no competing interests.

## Authors' contributions

CS - took care of the patient, drafted the manuscript; MA - took care of the patient, drafted the manuscript; AH - drafted the manuscript, checked for grammar and spelling; RS - performed gynaecologic examination, literature research; GW - took care of the patient, performed surgery, literature research; NW - performed pathologic and histologic analysis, drafted the manuscript; GB - performed pathologic and histologic analysis, drafted the manuscript; GM - performed radiologic examinations, drafted the manuscript; JF - performed neurologic examination, drafted the manuscript.

All authors read and approved the final manuscript.
